# CD36 Recruits α_5_β_1_ Integrin to Promote Cytoadherence of *P. falciparum*-Infected Erythrocytes

**DOI:** 10.1371/journal.ppat.1003590

**Published:** 2013-08-29

**Authors:** Shevaun P. Davis, Kristine Lee, Mark R. Gillrie, Lina Roa, Matthias Amrein, May Ho

**Affiliations:** 1 Department of Microbiology, Immunology and Infectious Diseases, University of Calgary, Calgary, Alberta, Canada; 2 Department of Anatomy and Cell Biology, University of Calgary, Calgary, Alberta, Canada; Seattle Biomedical Research Institute, United States of America

## Abstract

The adhesion of *Plasmodium falciparum*-infected erythrocytes (IRBC) to receptors on different host cells plays a divergent yet critical role in determining the progression and outcome of the infection. Based on our ex vivo studies with clinical parasite isolates from adult Thai patients, we have previously proposed a paradigm for IRBC cytoadherence under physiological shear stress that consists of a recruitment cascade mediated largely by P-selectin, ICAM-1 and CD36 on primary human dermal microvascular endothelium (HDMEC). In addition, we detected post-adhesion signaling events involving Src family kinases and the adaptor protein p130CAS in endothelial cells that lead to CD36 clustering and cytoskeletal rearrangement which enhance the magnitude of the adhesive strength, allowing adherent IRBC to withstand shear stress of up to 20 dynes/cm^2^. In this study, we addressed whether CD36 supports IRBC adhesion as part of an assembly of membrane receptors. Using a combination of flow chamber assay, atomic force and confocal microscopy, we showed for the first time by loss- and gain-of function assays that in the resting state, the integrin α_5_β_1_ does not support adhesive interactions between IRBC and HDMEC. Upon IRBC adhesion to CD36, the integrin is recruited either passively as part of a molecular complex with CD36, or actively to the site of IRBC attachment through phosphorylation of Src family kinases, a process that is Ca^2+^-dependent. Clustering of β_1_ integrin is associated with an increase in IRBC recruitment as well as in adhesive strength after attachment (∼40% in both cases). The adhesion of IRBC to a multimolecular complex on the surface of endothelial cells could be of critical importance in enabling adherent IRBC to withstand the high shear stress in the microcirculations. Targeting integrins may provide a novel approach to decrease IRBC cytoadherence to microvascular endothelium.

## Author Summary

Of the several species of malaria parasites that infect humans, disease caused by *Plasmodium falciparum* is responsible for most of the deaths. The unique pathological finding of this infection is the intense adhesion of infected red blood cells (IRBC) in the microcirculation, resulting in obstruction to blood flow and organ dysfunction. The focus of our research is to identify the molecules on host endothelial cells that support the adhesion as potential therapeutic targets. In this report, we showed for the first time a functional association between CD36, a well studied adhesion molecule for parasite adhesion, and α_5_β_1_, a member of the integrin family of adhesion molecules that are important for adhesion of leukocytes to blood vessels and cell adhesion to the extracellular matrix. We found that by itself, α_5_β_1_ integrin does not support IRBC adhesion. When IRBC adhere to CD36, the integrin is recruited to the site of adhesion through activation of the Src family kinase signaling pathway. As a result, both the number of adherent IRBC and the strength with which they adhere is greatly increased. These results demonstrate that IRBC adhesion is a dynamic and complex process. We need to identify each of the functional components to design anti-adhesive therapy.

## Introduction

Cell-cell interaction in the microvasculature is a complex process that involves multiple ligands and receptors that mediate different types of adhesive behavior in a sequential manner. The adhesive cascade is best studied in leukocyte-endothelial cell interactions that includes leukocyte tethering, crawling, rolling and adhesion on endothelium, followed by transmigration of leukocytes into extravascular tissues [1]. The strength of the interaction between ligands and receptors at each stage of the cascade can be qualitatively or quantitatively regulated by molecular events such as conformational change of the adhesion molecules, and/or intracellular signaling in both leukocytes and endothelial cells leading to modification of biological processes such as calcium flux, protein phosphorylation, cytoskeletal rearrangement and cell migration [2].

The adhesive interaction between *Plasmodium falciparum*-infected erythrocytes (IRBC) with vascular endothelium, the most consistent pathological finding in the human infection, is also governed by similar molecular mechanisms. Based on our findings with clinical parasite isolates obtained directly from infected patients, we have previously proposed a paradigm for IRBC cytoadherence under flow conditions that consists of a recruitment component that involves tethering, rolling and adhesion of IRBC that is mediated largely by P-selectin, ICAM-1 and CD36 respectively on primary human dermal microvascular endothelium (HDMEC) [3], [4]. In addition, we detected post-adhesion signaling events involving Src family kinases and the adaptor protein p130CAS in endothelial cells that lead to receptor clustering and cytoskeletal rearrangement which in turn enhance the magnitude of the adhesive strength, allowing adherent IRBC to withstand higher shear stress [5], [6]. Intracellular signaling in endothelial cell lines has also been shown for parasite lines and clones selected to adhere to ICAM-1 [7]. Together, these findings underscore the complexity of the cytoadherence process in the vasculature that might not be appreciated when studied as isolated ligand-receptor interactions on recombinant proteins or transfectants [8], [9], [10].

The class B scavenger molecule CD36 has a unique relationship with IRBC. CD36 is highly expressed in vital organs such as the lung, liver and kidney [11], and scantily in the brain [12]. CD36-mediated cytoadherence may contribute to dysfunction in these organs by impairing microcirculatory blood flow, although a direct link between IRBC adhesion to CD36 or any other receptor on primary endothelium under flow conditions has not been established. A recent report on *P. berghei* infection in mice suggests that CD36-dependent tissue sequestration may also promote parasite growth and other parasite survival benefits [13]. This long suspected association makes teleological sense as cytoadherence has likely evolved as a mechanism for host evasion. On the other hand, platelets have been shown to have a direct cytotoxic effect on IRBC adherent on CD36 through the release of platelet factor 4 (PF4) that binds to the Duffy blood group antigen on erythrocytes[14]. PF4 acts by its lytic activity on the food vacuole of the intraerythrocytic parasite while sparing the red cell membrane [15]. Collectively, these findings indicate that IRBC can interact with CD36 on different host cells with diverse biological effects.

An important question regarding IRBC–host cell interaction that has not been addressed is whether CD36 supports IRBC adhesion alone, or as part of an assembly of membrane receptors as it does in response to fibrillar α-amyloid [16], [17], [18]. The engagement and focal aggregation of the receptors following initial IRBC adhesion may lead to the formation of a functional complex which increases the strength of the adhesive interactions critical for determining adhesion in the microvasculature in vivo. IRBC could bind directly to multiple host surface molecules through different domains on the cytoadherent parasite ligand *Plasmodium falciparum* erythrocyte membrane protein 1 (PfEMP1) [19]. Alternatively, the involvement of other membrane receptors may occur downstream of CD36 ligation by being recruited to the site of adhesion where cross-talk between signaling molecules is facilitated [20]. In either scenario, integrins, a family of heterodimeric, non-covalently bound cell surface receptors, are likely candidate molecules to be involved, as they promote adhesion to other cells and matrix proteins, and are often associated physically and functionally with CD36 [21]. Indeed, CD36 is known to guide integrins into signaling rafts, and in so doing, may regulate integrin function. IRBC may bind to integrins directly through the tri-amino acid motif arginine-glycine-aspartic acid (RGD) present on PfEMP1 [22], [23], [24], or interact with integrins through binding to thrombospondin-1(TSP-1) [25]. In support of a role for integrins in cytoadherence, an anti-α_v_ antibody has been reported to partially inhibit the adhesion of a laboratory-adapted parasite line to HDMEC under flow conditions in vitro [26]. There is also evidence that IRBC and apoptotic leukocytes could downregulate dendritic cell function through CD36 or α_v_β_3_ [27]. As over 250 anti-integrin drugs have now entered clinical trials [28], an understanding of the role of integrins in cytoadherence may lead to the novel application of some of the available agents as adjunctive therapy in the treatment of severe falciparum malaria.

In this study, we used a combination of flow chamber assay, atomic force microscopy (AFM) and confocal microscopy to investigate the role of integrins in cytoadherence. Our results reveal a novel and robust co-operative association of CD36 with the integrin α_5_β_1_ in microvascular endothelium as a result of Src family kinase signaling. The formation of an IRBC-endothelial cell synapse consisting of multiple adhesion molecules contributes significantly to overall IRBC recruitment and adhesive strength. Identification of the dynamic interactions among all binding partners for IRBC on primary endothelial cells will provide a theoretical basis for the rational design of anti-adhesive therapy.

## Results

### Expression of integrins on HDMEC

A number of integrins are expressed on microvascular endothelial cells [29], including α_v_β_3_, α_v_β_1_, α_2_β_1_, α_3_β_1_ and α_5_β_1_ that recognize RGD motifs on their ligands [30]. In this study, we focused on α_v,_ α_5_ and β_1_ that have been shown to be associated with CD36 on HDMEC by both an antibody array and by co-immunoprecipitation [31]. All 4 proteins were shown to be surface expressed by flow cytometry (Figure S1A) and by confocal immunofluorescence microscopy (Figure S1B). The latter findings indicate that the integrin molecules are expressed on the luminal surface of endothelial monolayers and are therefore available for interaction with IRBC.

### RGD peptide inhibits cytoadherence under physiological shear stress

**Figure 1:**
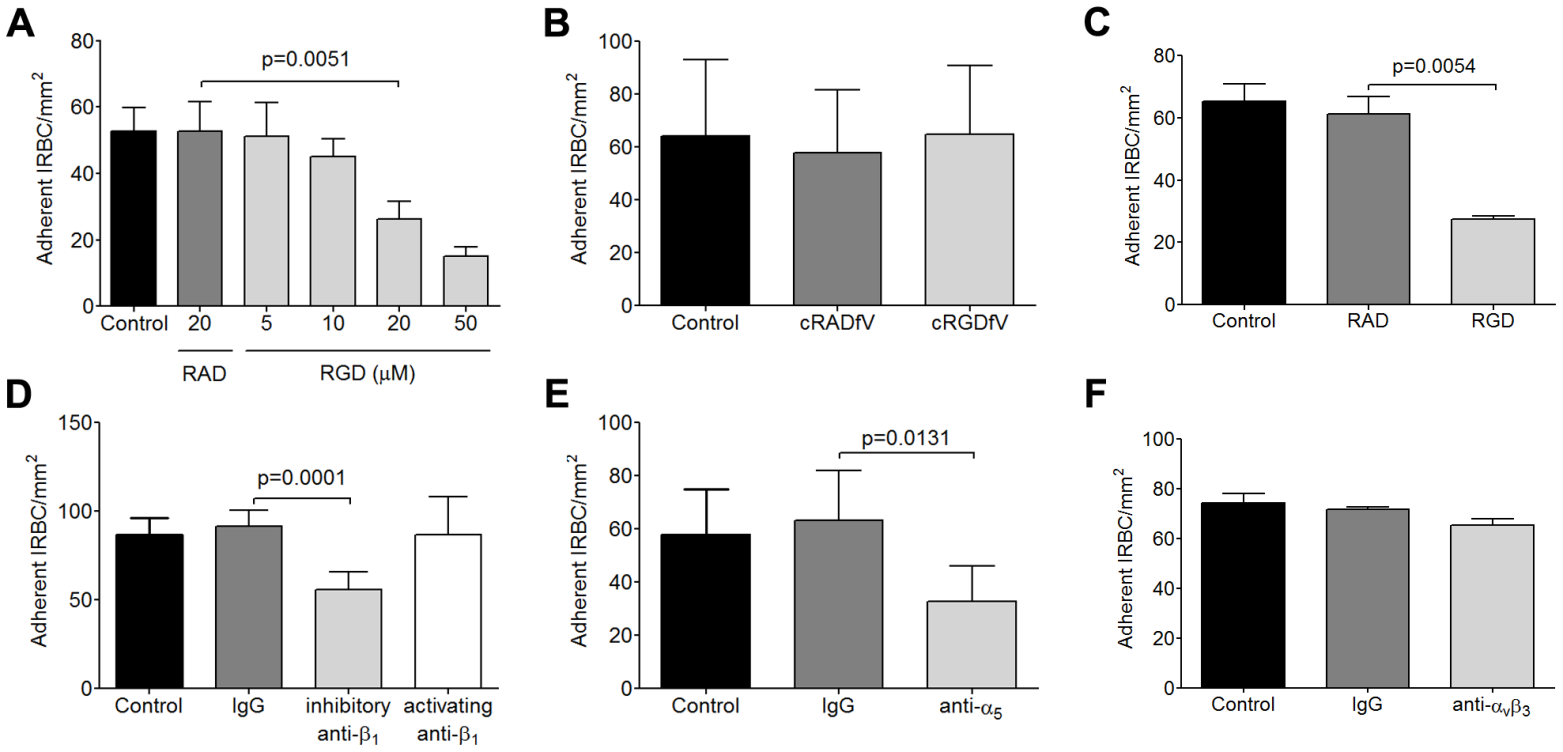
Inhibition of cytoadherence on HDMEC by RGD peptide and anti-b_1_ and anti-a_5_ mAb. (A) Adhesion of IRBC to endothelial monolayers pre-incubated with 20 µM RAD or 5–50 µM RGD peptide for 30 min at 37°C in 5% CO_2_. A 1% hematoctit suspension of IRBC from the lab-adapted parasite clone 7G8 was drawn over the monolayers at 1 dyne/cm^2^. Results shown are the mean number of adherent IRBC/mm^2^ in 4–6 microscopic fields (20×) after 7 min of infusion (n = 6). (B) Adhesion of 7G8 parasites to endothelial monolayers pre-incubated with 20 µM cRADfV or cRGDfV peptide (n = 3). (C) Adhesion of 3 clinical parasite isolates to endothelial monolayers pre-incubated with 20 µM RAD or RGD peptide (n = 10 for 3 clinical isolates each tested in 3 to 4 independent experiments). (D) Adhesion of clinical isolates to endothelial monolayers pre-incubated with control IgG_1_, an inhibitory anti-β_1_ integrin mAb TDM29 or the activating anti-β_1_ integrin mAb TS2/16 at 10 mg/ml (n = 3 for 3 clinical isolates each tested in 1 independent experiment). (E) Adhesion of clinical isolates to endothelial monolayers pre-incubated with control IgG_1_, and an inhibitory anti-α_5_ integrin mAb JBS5 at 10 µg/ml (n = 3 for 3 clinical isolates each tested in 1 independent experiment). (F) Adhesion of clinical isolates to endothelial monolayers pre-incubated with control IgG_1_, and an inhibitory anti-α_v_β_3_ integrin mAb 23C6 at 10 µg/ml (n = 3 for 3 clinical isolates each tested in 1 independent experiment).

To begin to assess a role for integrins in IRBC adhesion, HDMEC monolayers were pretreated with 5 to 50 µM of the integrin antagonist RGD (H-Gly-Arg-Gly-Asp-Ser-Pro-OH) for 30 min at 37°C before the infusion of IRBC in a flow chamber assay. The IRBC used was a lab adapted clone 7G8 that binds to both CD36 and ICAM-1. The results indicate that RGD inhibited IRBC adhesion in a dose-dependent manner (Figure 1A), while the control peptide RAD (H-Gly-Arg-Ala-Asp-Ser-Pro-OH) had no effect. The decrease in the total number of adherent IRBC on RGD treated monolayers was associated with an increase in the number of rolling cells that did not adhere (control 52±8 vs RGD 95±12 cells/7 min, n = 6, p = 0.0034), suggesting that integrins are critical for firm adhesion to occur.

It has been previously reported that an anti-α_v_ antibody inhibited IRBC adhesion to HDMEC [26]. The integrin was assumed to be α_v_β_3_ by the authors and in subsequent publications [24], [32], [33], but the finding has never been confirmed. To determine if α_v_β_3_ could mediate IRBC adhesion, we pre-incubated HDMEC with the cylic peptide cRGDfV (cyclo(-Arg-Gly-Asp-D-Phe-Val)) that is a specific inhibitor for the α_v_β_3_ and α_v_β_5_ integrins [34]. In contrast to RGD, the cyclic peptide at 20 µM had no effect on cytoadherence of 7G8 parasites (Figure 1B).

To confirm that the ability of IRBC to interact with integrins is not acquired as a result of prolonged passage in the laboratory, flow chamber experiments were performed with clinical parasite isolates. As in the case with 7G8, adhesion of cryopreserved IRBC from acutely infected patients, cultured for 24 to 36 h, was inhibited by RGD at 20 µM (Figure 1C). Moreover, cytoadherence of the clinical isolates was reduced by the inhibitory anti-β_1_ mAb TDM29 (10 µg/ml) but not the activating anti-β_1_ mAb TS2/16 (10 µg/ml) (Figure 1D). Inhibition of adhesion was also seen with the anti-α_5_ mAb JBS5 (10 µg/ml) (Figure 1E), suggesting that binding of IRBC is to an epitope involving both subunits of the heterodimer. Consistent with the results obtained with cRGDfV, an inhibitory anti-α_v_β_3_ mAb 23C6 (10 µg/ml) did not affect cytoadherence (Figure 1F).

### RGD peptide and integrin-specific Abs inhibit adhesive strength by AFM

**Figure 2:**
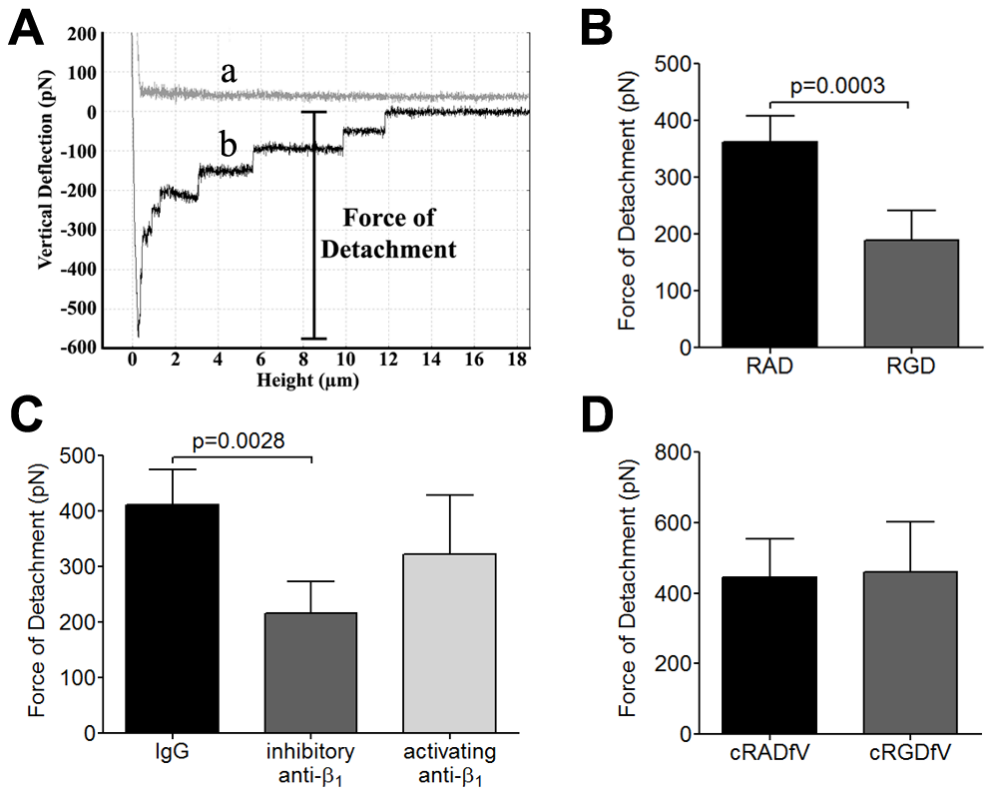
Inhibition of adhesive strength between IRBC and HDMEC by RGD peptide and anti-β_1_ mAb. (A) Schematic representation of a typical AFM force curve which depicts a) approach of the IRBC to an endothelial monolayer and b) retraction of the IRBC. The bar indicates the force of detachment that is used as a measure of adhesive strength between IRBC and endothelium. (B) Force measurement on endothelial monolayers pre-incubated with 20 µM RAD or RGD peptide for 30 min at 37°C in 5% CO_2_ (n = 7). (C) Force measurement on endothelial monolayers pre-incubated with control IgG_1_, an inhibitory anti-β_1_ integrin mAb TDM29 or the activating anti-β_1_ integrin mAb TS2/16 at 10 µg/ml (n = 4). (D) Force measurement on endothelial monolayers pre-incubated with 20 µM cRADfV or cRGDfV peptide (n = 3). For each experiment, 2 IRBC were brought into contact with 3 HDMEC. Contact for 5 min was maintained with a constant force of 150 pN.

We next used atomic force microscopy (AFM) to directly measure the adhesive force between IRBC and HDMEC at the single cell level as previously described [6]. An IRBC attached to the cantilever by dopamine hydrochloride was brought into contact with an endothelial cell using a contact force of 150 pN at a steady velocity of 1.5 µm sec^−1^. These parameters were chosen because they approximate the marginating force experienced by a more rigid IRBC as it is pushed from centreline blood flow towards the vessel wall by NRBC. Upon contact, the IRBC and HDMEC were allowed to remain in contact for 5 min before it was withdrawn at the same velocity. Adhesive strength was measured as the force required for detachment of the IRBC from an HDMEC (Figure 2A). In a previous study, we found that the detachment force between IRBC and HDMEC increased rapidly with the duration of contact, so that the magnitude of the force could be 5 to 6 fold higher by the end of 5 min [6]. The resulting increase in adhesion strength enabled adherent IRBC to withstand shear stress of up to 20 dynes/cm^2^.

To determine if integrins contribute to this enhancement of adhesive force following contact, force measurements were performed on HDMEC monolayers pre-treated with RAD or RGD using 7G8 parasites. The results indicate that RGD (Figure 2B) and the inhibitory anti-β_1_ mAb TDM29 (Figure 2C) both inhibited adhesive force of IRBC by approximately 40% while RAD or the activating anti-β_1_ mAb TS2/16 had no effect. As in the case of adhesion in the flow chamber assay, cRGDfV had no effect on detachment force (Figure 2D). Together, our results indicate a significant role for integrins both in the firm adhesion of IRBC under flow conditions and in the post-contact increase in adhesive strength between IRBC and endothelial cells.

### RGD peptide inhibits cytoadherence and adhesive strength of IRBC to TNF-α-stimulated HDMEC

**Figure 3:**
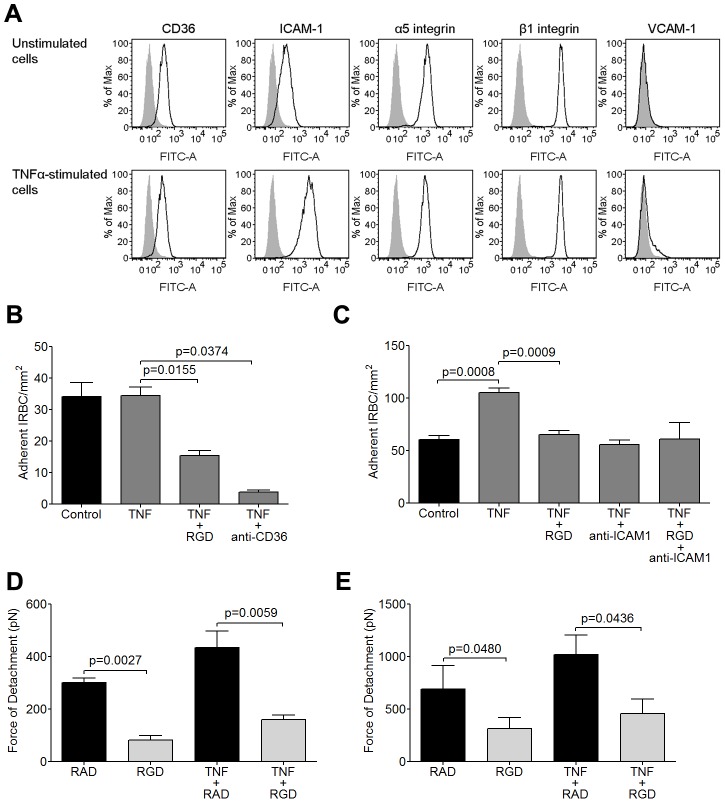
Inhibition of IRBC cytoadherence and adhesive force on TNF-α-stimulated HDMEC by RGD peptide. (A) Flow cytometric analysis of the expression of CD36, ICAM-1, α_5,_ β_1_ and VCAM-1 on unstimulated and TNF-α-stimulated HDMEC. TNF-α was added at 1 ng/ml for 20–24 h. Results shown are representative of at least 3 experiments. (B) Adhesion of IRBC from the parasite line 7G8 to unstimulated and TNF-α-stimulated endothelial monolayers pre-incubated with 20 µM RGD peptide or 5 µg/ml of anti-CD36 for 30 min at 37°C in 5% CO_2_ (n = 3). (C) Adhesion of IRBC from two clinical parasite isolates to unstimulated and TNF-α-stimulated endothelial monolayers pre-incubated with 20 µM RGD peptide, 5 µg/ml of anti-ICAM-1 or both (n = 5 with 2 clinical isolates). (D) Force of detachment measurements of 7G8 parasites on unstimulated and TNF-α-stimulated endothelial monolayers pre-incubated with 20 µM RAD or RGD peptide (n = 3). (E) Force of detachment measurements of two clinical parasites on unstimulated and TNF-α-stimulated endothelial monolayers pre-incubated with 20 µM RAD or RGD peptide (n = 3 with 2 clinical isolates).

As cytoadherence occurs in a proinflammatory environment during acute *P. falciparum* infection [35], [36], the inhibitory effect of RGD on IRBC adhesion in the flow chamber assay was studied on HDMEC monolayers that had been pre-stimulated with 1 ng/ml of TNF-α for 20 to 24 h. The results are shown in Figure 3. In accordance with what we reported previously [3], TNF-α stimulation of HDMEC upregulated ICAM-1 expression, and induced VCAM-1 in a small percentage of cells (Figure 3A). The expression of CD36, α_5_ and β_1_ remained essentially unchanged. Stimulation of HDMEC with TNF-α at 1 ng/ml did not lead to an increase in the number of adherent IRBC of the 7G8 parasite line (Figure 3B). IRBC adhesion was reduced by RGD as on unstimulated endothelium, and was inhibited by >90% by the anti-CD36 mAb FA6.

We next tested two of the three clinical isolates that were studied in Figure 1. IRBC adhesion increased by almost two fold on cytokine-stimulated HDMEC (Figure 3C). Adhesion was inhibited to a similar extent by both RGD and by an anti-ICAM-1 mAb. However, there was no additive effect when monolayers were pre-treated with a combination of RGD and anti-ICAM-1.

The effect of TNF-α stimulation on the strength of IRBC adhesion was investigated by AFM. For the 7G8 parasite line as well as the 2 clinical isolates, there was no difference in the magnitude of the adhesive force generated over 5 minutes on TNF-α-stimulated endothelium compared to unstimulated controls (Figure 3D). As well, pre-incubation with RGD inhibited _5_adhesive strength as on undstimulated endothelium. Collectively, the results suggest that integrins contribute to both an increase in IRBC adhesion and adhesive strength on cytokine-stimulated HDMEC.

### siRNA knockdown of β_1_ and α_5_ integrins

**Figure 4:**
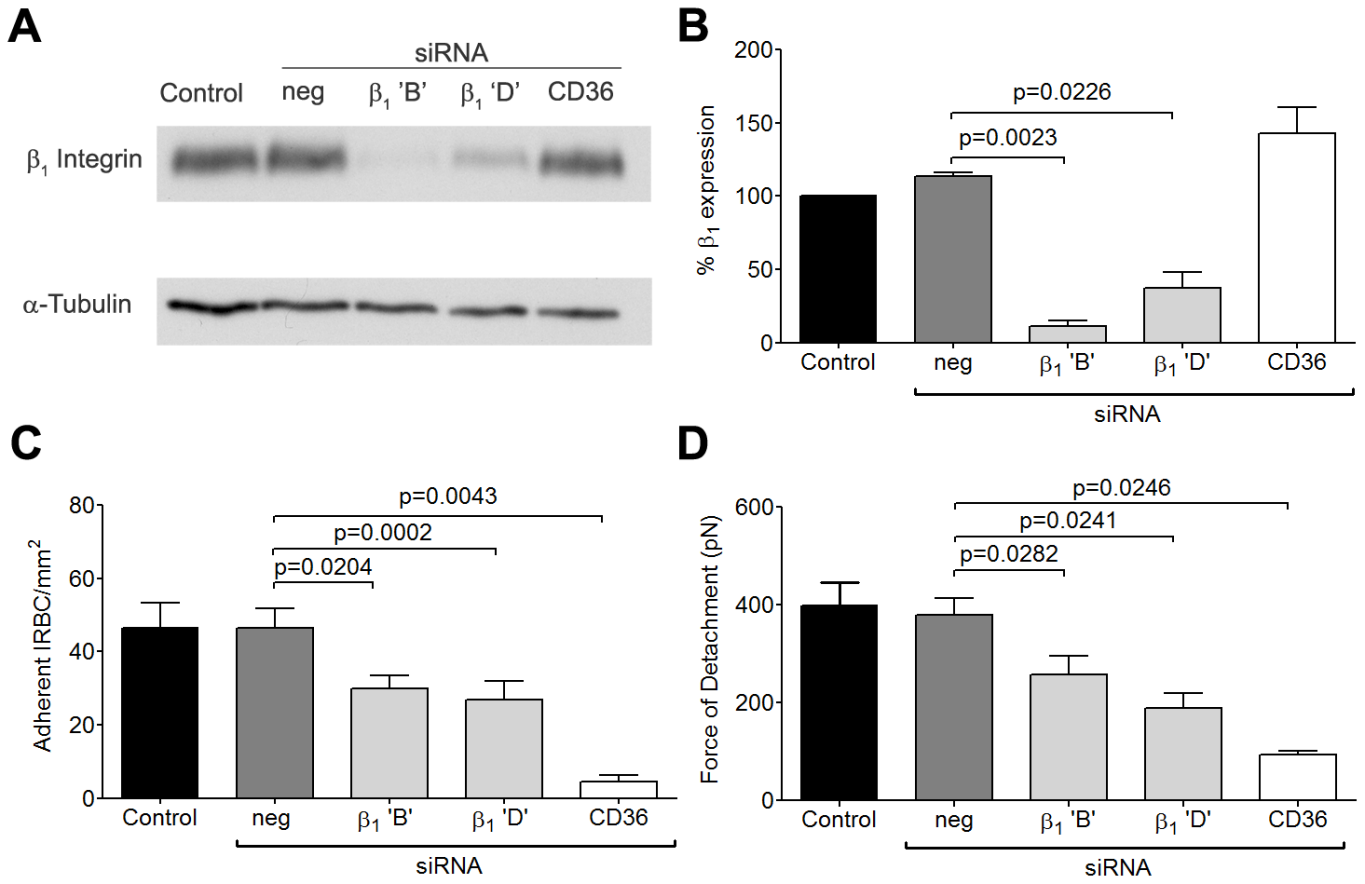
Inhibition of cytoadherence and adhesive strength on HDMEC transfected with small interference RNA of β_1_ integrin. (A) Western blot analysis of HDMEC lysates 72 h after transfection with 20 nM of negative siRNA and siRNA for β_1_ integrin ‘B’ and ‘D’, and CD36. Blots were probed with mAb anti-β_1_ integrin TS2/16 (top) and anti-α-tubulin (bottom). Results shown are representative of 3 independent experiments. (B) Densitometric analysis showing the effectiveness of the knockdown of β_1_ integrin (n = 3). (C) Adhesion of IRBC to β_1_ integrin and CD36 knock down endothelial monolayers (n = 3). (D) Force of detachment for IRBC on β_1_ integrin and CD36 knock down endothelial monolayers (n = 4).

**Figure 5:**
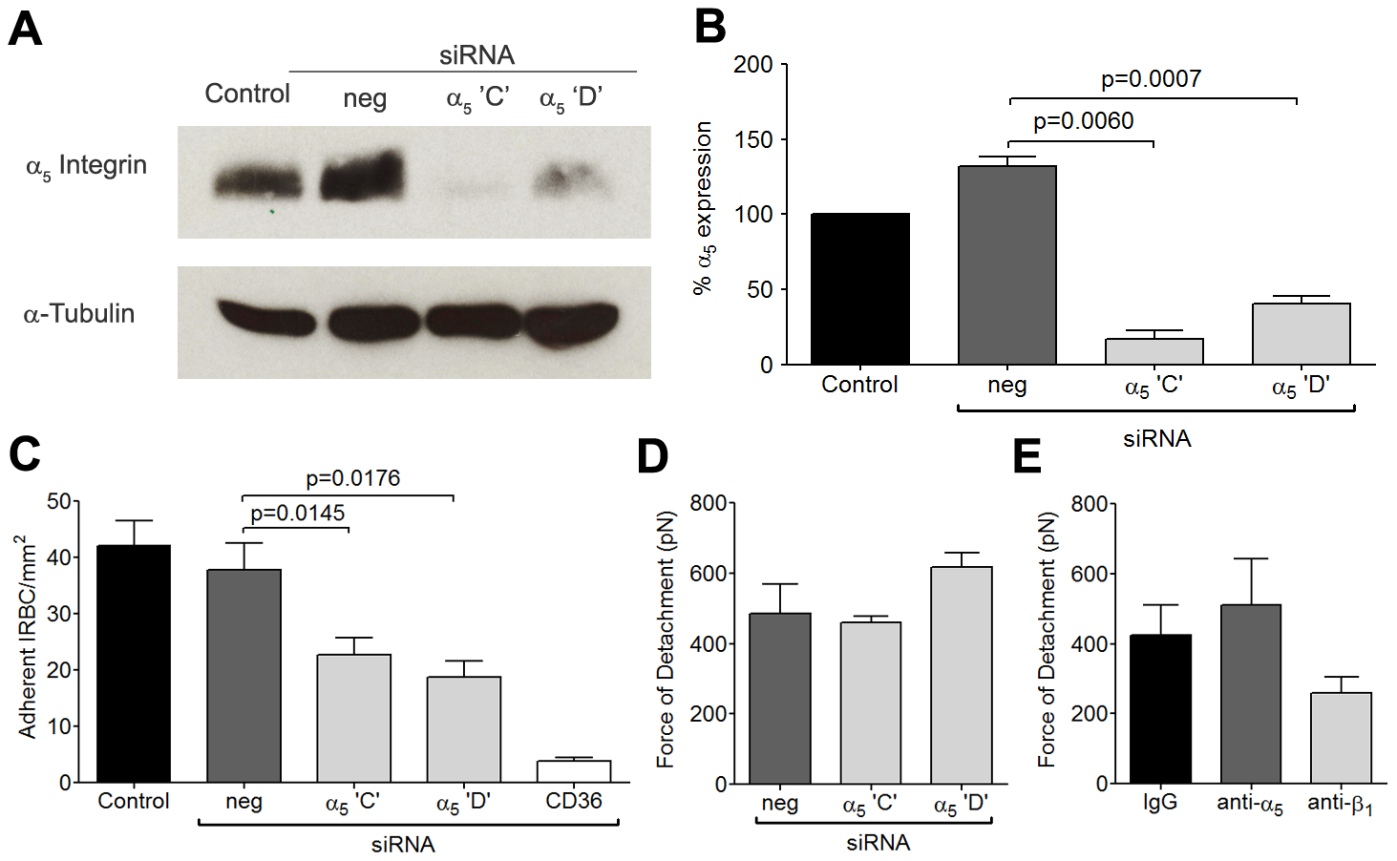
Inhibition of cytoadherence but not adhesive strength on HDMEC transfected with small interference RNA of α_5_ integrin. (A) Western blot analysis of HDMEC lysates 72 h after transfection with 20 nM of negative siRNA and siRNA for α_5_ integrin ‘C’ and ‘D, and CD36. Blots were probed with a polyclonal anti-α_5_ integrin antibody (top) and a monoclonal anti-α-tubulin antibody (bottom). Results shown are representative of 3 independent experiments. (B) Densitometric analysis showing the effectiveness of the knockdown of α_5_ integrin (n = 3). (C) Adhesion of IRBC to α_5_ integrin knockdown endothelial monolayers (n = 3). (D) Force of detachment for IRBC on α_5_ integrin knockdown endothelial monolayers (n = 2). (E) Force of detachment for IRBC on endothelial monolayers pre-incubated with the anti-α_5_ mAb JBS5 (n = 2). Results for (D) and (E) are shown as mean ± SD.

To more specifically assess the role of β_1_ integrin in the increase in IRBC recruitment and the adhesive strength of the IRBC-integrin interaction, we performed gene knock down of β_1_ integrin in HDMEC by siRNA. Loss of β_1_ integrin protein production was confirmed by Western blot (Figure 4A and B). The targeted deletion of β_1_ integrin did not alter endothelial CD36 or ICAM-1 expression (data not shown). The loss of β_1_ integrin led to a reduction in IRBC adhesion in the flow chamber assay (Figure 4C) as well as the adhesive strength as measured by AFM (Figure 4D). These results confirmed a functional role for β_1_ integrin in mediating IRBC cytoadherence. Interestingly, when the α_5_ integrin was similarly knocked down (Figure 5A and B), a reduction in IRBC adhesion in the flow chamber assay was observed (Figure 5C) while adhesive strength by AFM was unaffected (Figure 5D). The lack of effect of α_5_ knock down on adhesive force was consistent with the inability of the mAb JBS5 to inhibit adhesive force (Figure 5E).

### IRBC binds to α_5_β_1_ only in the presence of CD36

**Figure 6:**
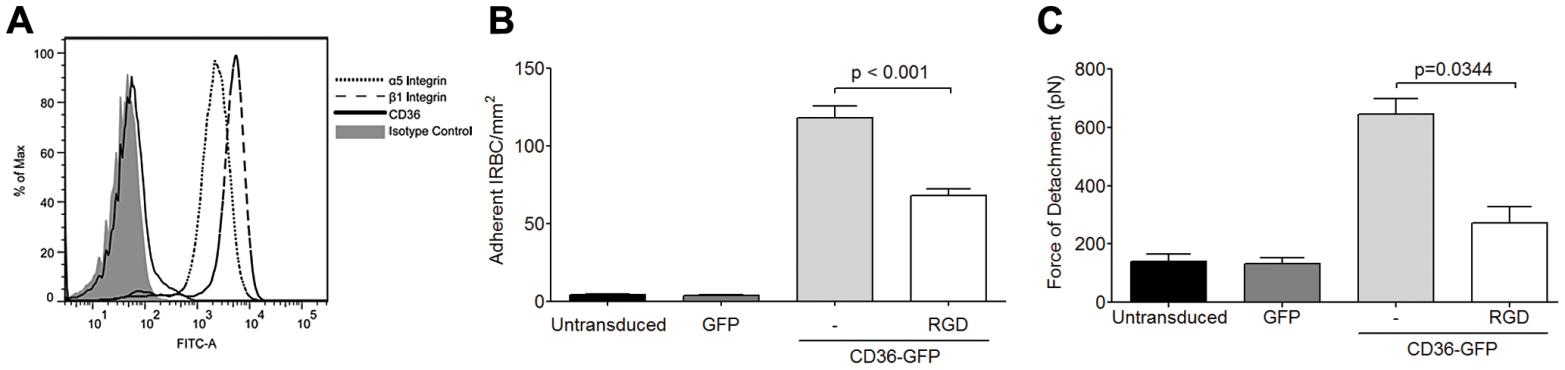
Adhesion of IRBC on the endothelial cell line HMEC-1. (A) Expression of the integrins α_5_ and β_1_ but not CD36 on HMEC-1. Results are representative of 3 independent experiments. (B) Adhesion of IRBC to untransduced, GFP-transduced and CD36-GFP transduced HMEC-1 (n = 3). (C) Force of detachment for IRBC on GFP-transduced and CD36-GFP transduced HMEC-1 (n = 3).

The inhibitory effect of an antagonist peptide and mAb to the integrin α_5_β_1_ suggest that IRBC may be binding directly to the integrin. Indeed, RGD motifs have been demonstrated in several DBL domains of the cytoadherent ligand PfEMP1 in a number of parasite lines [22], [23], [24]. These parasite RGD motifs could potentially interact with endothelial integrins and contribute to the overall adhesive force between IRBC and endothelium. To determine if IRBC can bind to α_5_β_1_ under flow conditions, IRBC were infused over HMEC-1 monolayers. This endothelial cell line, derived from HDMEC, does not express CD36 [29], or only at a very low level (Figure 6A). In contrast, the α_5_ and β_1_ integrins are highly expressed (Figure 6A). We found that <5 IRBC/mm^2^ rolled and/or adhered on HMEC-1 for the total duration of a 7-min infusion in the flow chamber assay (Figure 6B). However, when HMEC-1 was tranduced with CD36-GFP, but not GFP alone, there was a dramatic increase in IRBC adhesion that was reduced by pre-treatment of the monolayer with RGD. A similar effect of CD36 on β_1_ integrin function was detected by AFM, as indicated by an increase in adhesive force on CD36 transduced cells that was partially inhibited by pre-treatment of the monolayer with RGD (Figure 6C). These results suggest that in its resting state, β_1_ integrin by itself is unable to support IRBC adhesion under flow conditions. The presence of CD36 may lead to proadhesive changes of β_1_ integrin through changes in i) integrin surface expression, ii) conformation or iii) subcellular localization.

### Integrin clustering at site of IRBC adhesion is CD36-dependent

To determine the mechanism of CD36-β_1_ integrin interaction with respect to cytoadherence, we first determined if the presence of CD36 modulated surface expression of β_1_ integrin using HMEC-1 cells. No increase in β_1_ integrin expression on HMEC-1 was seen by flow cytometry after the transduction of CD36 (data not shown). The contribution of conformational changes was also unlikely as neither the activating β_1_ antibody TS2/16 (Figure 1D) nor the addition of 1 mM Mn^2+^ (data not shown), both of which activate β_1_ integrin [37], had any effect on IRBC adhesion. The most likely mechanism is integrin clustering. Indeed, clustering of α_5_β_1_ in mouse embryonic fibroblasts as a result of lateral diffusion has been demonstrated to increase adhesive strength between the integrin and its natural ligand fibronectin, while clustering of α_v_β_3_ contributes to mechanotransduction [38].

**Figure 7:**
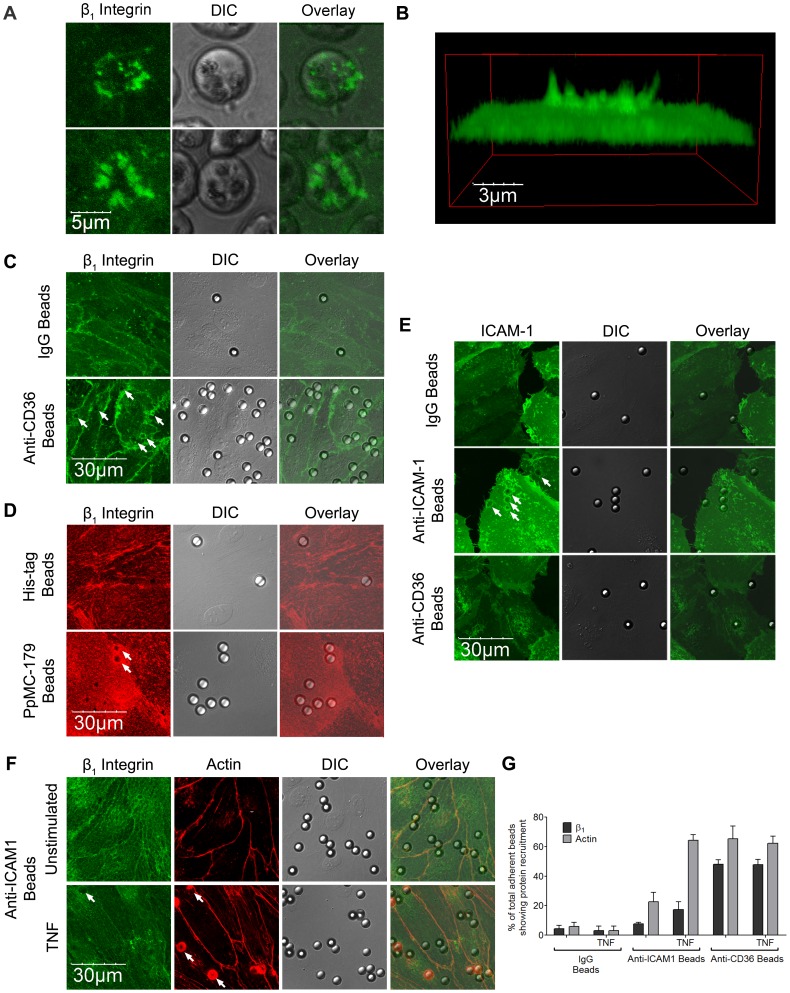
β_1_ integrin recruitment by adherent IRBC, antibody- or PpMC-179 coated-beads. HDMEC were grown to 95% confluence in ibidi VI chambers. (A) IRBC purified on a MACS separation column were added to a monolayer at 0.1% hematocrit. An FITC-labelled anti-b1integrin mAb was added at 10 µg/ml. The IRBC interaction with HDMEC was imaged in a humidified chamber with 5% CO_2_ at 37°C. Results are representative of 2 experiments. (B) 3D reconstruction of the cup-shaped structure formed by clustered β_1_ integrin on the endothelial cell membrane. (C and D) HDMEC monolayers in ibidi VI chambers were incubated with beads coated with IgG_1_, anti-CD36, anti-HIS or PpMC-179, the CD36 binding peptide of PfEMP1, for 30 min at 37°C in 5% CO_2_. The monolayers were washed 2× with HBSS to remove unbound beads prior to fixing with 1% PFA for 30 minutes at room temperature. Fixed cells were stained with an anti-β_1_ mAb at 10 µg/ml followed by Alexa 488-labelled anti-mouse IgG_1_ (C) or with a PE-labelled anti-β_1_ integrin mAb at 10 mg/ml (D). Results shown are representative of 3 experiments. (E) HDMEC monolayers in ibidi chambers transduced with GFP-ICAM-1 were incubated with beads coated with IgG_1_, anti-ICAM-1 and anti-CD36, and processed as in (C) and (D). (F) Unstimulated and TNF-α-stimulated HDMEC monolayers in ibidi chambers were incubated with beads coated with IgG_1_, anti-ICAM-1 and anti-CD36, and processed as in (C) and (D). The actin cytoskeleton was visualized by permeabilizing the cells for 5 min with 0.2% TX-100 prior to adding 1 µl of rhodamine-phalloidin in 60 µl HBSS to each chamber for 30 min at room temperature. Results shown are for anti-ICAM-1 coated beads only, and are representative of 3 experiments. All images were taken on an Olympus IX81 inverted confocal microscope (Center Valley, Pa) with Fluorview 1000 acquisition software using a PlanAPO 60× N.A. 1.42 oil immersion objective. White arrows indicate representative sites of β_1_ integrin or ICAM-1 recruitment. (G) Quantification of microscopic changes seen in (F). For every condition in every experiment, three randomly selected microscopic fields at 60× magnification were selected. Each field with 15–25 adherent beads were scanned, and adherent beads associated with protein recruitment were scored as positive. Beads partially in the field of view or rings with no adherent beads were excluded in the enumeration. Results are expressed as positive adherent beads/total adherent beads ×100% (n = 3).

To determine if β_1_ integrin is recruited upon IRBC adhesion, IRBC purified on a magnetic bead separation column (Miltenyi Biotec,Auburn, CA) were allowed to adhere to HDMEC. A labeled anti-β_1_ antibody was added and endothelial cells were observed by live cell imaging. Figure 7A shows that β_1_ integrin was recruited to the site of IRBC adhesion within minutes of the adhesion process. Moreover, the recruited β_1_ integrin forms a cup-shaped protrusion around the IRBC on the cell surface (Figure 7B). Interestingly, β_1_ integrin clustering was also seen with polystyrene beads coated with an anti-CD36 mAb FA6 (Figure 7C), or the recombinant PpMC-179 peptide representing the minimal binding domain of PfEMP1 (residues 88–267 of the CIDR domain of the parasite strain Malayan Camp) for CD36 [39] (Figure 7D). The results suggest that β_1_ integrin recruitment likely occurred as a result of CD36 ligation, either passively as a member of a molecular complex with CD36, or as a result of recruitment downstream of CD36 ligation. The recruitment of β_1_ integrin by CD36 was specific, as anti-CD36 coated beads did not recruit ICAM-1 on HDMEC transduced with GFP-ICAM-1 (Figure 7E). Compared to anti-CD36 coated beads, anti-ICAM-1 coated beads recruited much less β_1_ on TNF-α-stimulated endothelium (Figure 7F and 7G).

**Figure 8:**
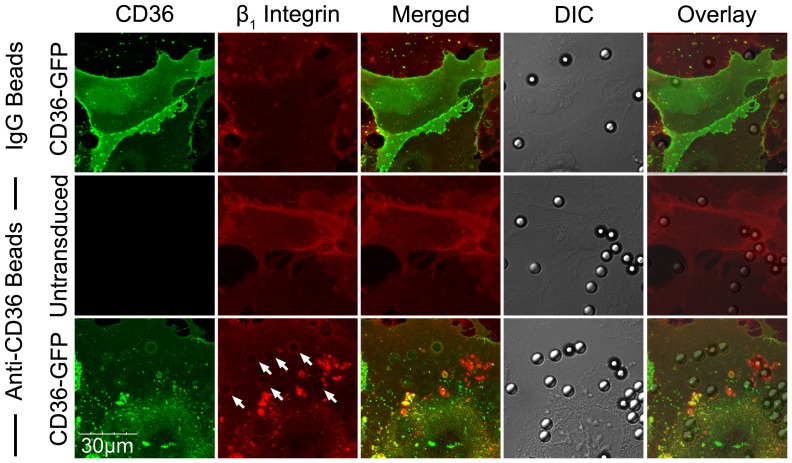
β_1_ integrin recruitment by adherent anti-CD36 coated beads on HMEC-1. HMEC-1 monolayers in ibidi chambers transduced with GFP-CD36 were incubated with beads coated with IgG_1_ or anti-CD36 for 30 min at 37°C in 5% CO_2_. The slides were fixed and stained as described in Figure 7 using a PE-labelled anti-β_1_ integrin mAb. Results shown are representative of 3 independent experiments.

The dependence of integrin clustering on CD36 was further demonstrated with HMEC-1 cells. When FA6-coated beads were added to HMEC-1, no β_1_ integrin clustering was seen on confocal microscopy (Figure 8). In contrast, CD36 and integrin clustering at the site of adhesion was clearly observed after HMEC-1 were transduced with CD36-GFP.

### Integrin α_5_β_1_ recruitment is mediated by Src family kinases and is calcium-dependent

**Figure 9:**
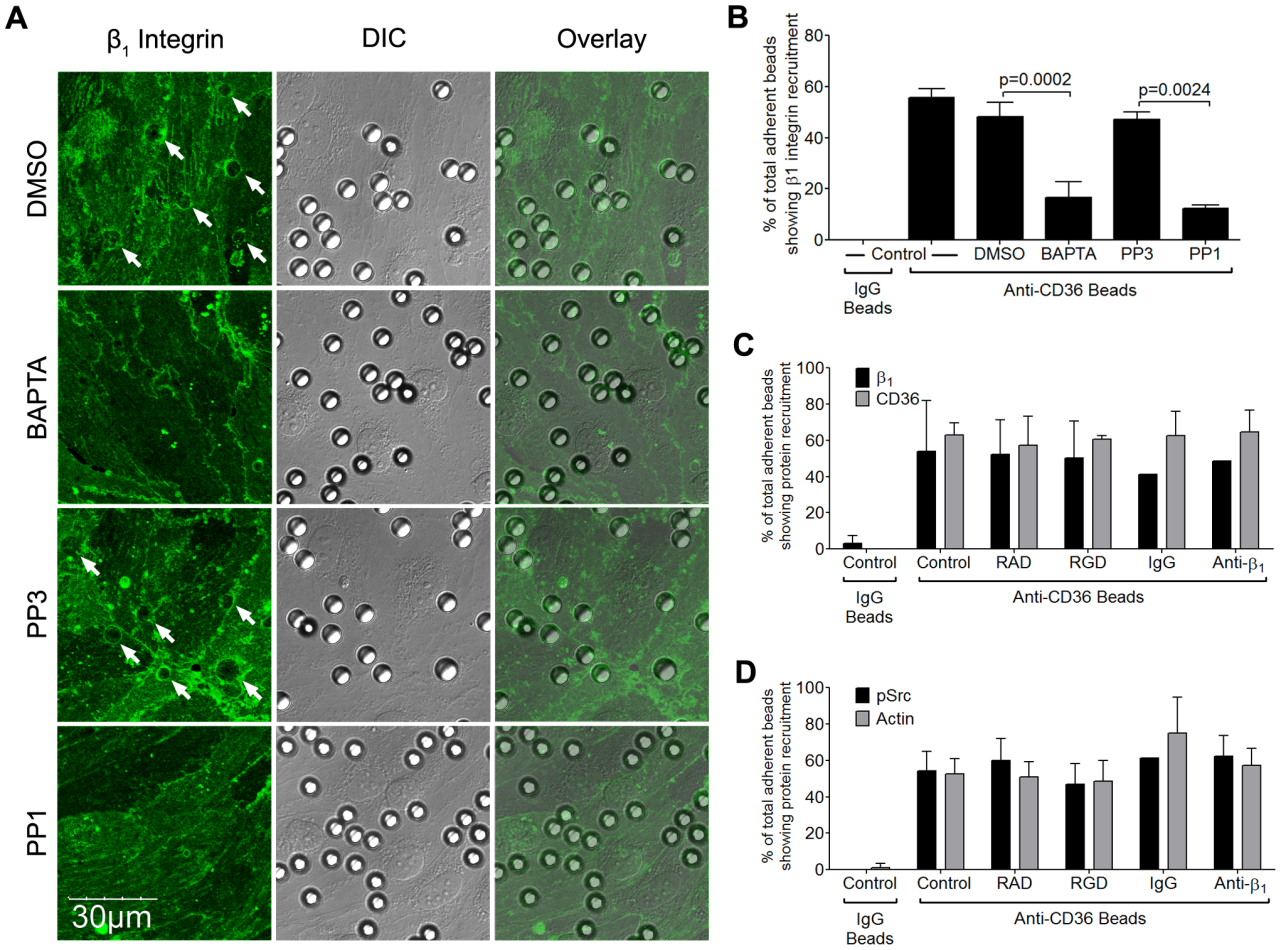
Inhibition of β_1_ integrin recruitment by Src family kinase inhibitor and intracellular Ca^2+^ chelator. (A) HDMEC monolayers were pre-incubated with PP3 or PP1 at a concentration of 10 µM for 2 hours, or DMSO or BAPTA for 30 min followed by HBSS for 30 min, at 37°C in 5% CO_2_. Anti-CD36 coated beads were then added to the monolayers for 30 min. After unattached beads were washed off, the monolayers were fixed and stained with Alexa 488-labeled anti-β_1_ integrin for 1 hr at room temperature. The images were taken as in Figure 7. Results shown are representative of 3 experiments. (B) Quantification of microscopic changes as described in Figure 7F. (C) HDMEC transduced with GFP-CD36 were either untreated, or pre-incubated with 20 µM RAD/RGD, or IgG_1_/inhibitory anti-β_1_ integrin mAb (clone TDM29) at 10 µg/ml for 30 min at 37°C in 5% CO_2_. After the addition of anti-CD36 coated beads for 30 min, the monolayers were fixed and stained as in (A). Quantification of 2 experiments is shown. (D) Anti-CD36 coated beads were added to peptide or antibody-treated HDMEC monolayers as in (C). The monolayers were fixed, permeabilized with 0.2%TX-100 for 5 min, and blocked with 1%BSA+0.003%TX-100 for 30 min. Monolayers were stained with a polyclonal anti-phospho-Src antibody overnight at 4°C followed by goat-anti-rabbit IgG-Alexa 488 for 1 hr at room temperature. The actin cytoskeleton was visualized by adding 1 µl of rhodamine-phalloidin in 60 µl HBSS to each chamber for 30 min at room temperature. Quantification of 2 experiments is shown.

If α_5_β_1_ clustering occurs downstream of CD36 ligation by IRBC, one would expect that the process would be mediated by Src family kinases that are activated by the binding of IRBC to CD36 [5], [6]. The possibility was investigated with anti-CD36 coated beads using PP1, a specific Src family kinase inhibitor and its inactive analog PP3. The requirement for Ca^2+^ was also investigated by preincubating HDMEC with the intracellular calcium chelator BAPTA-AM. Similar to the effects of the inhibitors on CD36 and actin recruitment [6], β_1_ integrin recruitment in response to anti-CD36 coated beads was significantly reduced by these inhibitors (Figure 9A and B). In contrast, neither RGD nor anti-β_1_ antibody had any effect on the recruitment of β_1_ integrin or CD36 (Figure 9C), or actin and phosphorylated Src (Figure 9D). Consistent with these observations, neither RGD nor anti-β_1_ mAb had any effect on the force of detachment of anti-CD36 coated beads as measured by AFM (data not shown). Together, these results indicate that β_1_ integrin clustering occurs as a downstream event of CD36 ligation and subsequent signaling events, and not through extracellular integrin activation.

### β_1_ integrin clustering increases the binding avidity with IRBC

**Figure 10:**
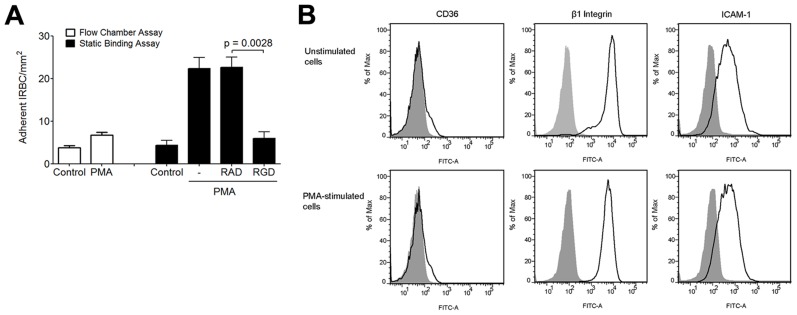
Effect of PMA on IRBC adhesion to HMEC-1. (A) Adhesion of IRBC on untreated HMEC-1 or HMEC-1 pre-incubated with PMA 50 ng/ml for 30 min at 37°C and 5%CO_2_ under flow (left) or static (right) conditions. In the static binding experiments, monolayers were pre-treated with either 50 ng/ml of PMA alone, or 100 µM RGD or RAD peptide was added after 10 min of incubation. At the end of 30 min, 1 ml of a 1% hematocrit suspension of IRBC in RPMI at 5–9% parasitemia was added and allowed to adhere for 20 min. The 35 mm dish with the HMEC-1 monolayer and IRBC was then mounted into the flow chamber system. HBSS was infused at 1 dyne/cm^2^, and the number of adherent cells were counted at 30 sec intervals for a total of 4 min and the mean taken (n = 3). (B) CD36, β_1_ integrin and ICAM-1 expression on control and PMA-treated HMEC-1 cells. Results are representative of 2 independent experiments.

Lateral diffusion of the β_2_ integrin LFA-1, as detected by single molecule tracking, can be induced by phorbol-12-myristate-13-acetate (PMA) and low dose cytochalasin D in lymphocytes [40] and monocytes [41]. The resulting integrin clustering leads to an increase in the number of adherent leukocytes on ICAM-1. To determine if PMA could also affect integrin interaction with IRBC, we performed the flow chamber assay with HMEC-1 monolayers with or without pre-treatment with PMA (50 ng/ml) for 30 min at 37°C. HMEC-1 cells were utilized in these experiments to allow for an assessment of IRBC adhesion directly to integrins in response to PMA in the absence of CD36. Fewer than 5 IRBC adhered on either untreated or treated monolayers under flow conditions (Figure 10A). However, PMA treatment significantly increased the number of IRBC that adhered in a static binding assay. In these experiments, monolayers were pre-treated with either 50 ng/ml of PMA alone for 30 min at 37°C, or 100 µM RGD or RAD peptide added after 10 min of incubation. At the end of 30 min, 1 ml of a 1% hematocrit suspension of IRBC in RPMI at 5–9% parasitemia was added and allowed to adhere for 20 min. The 35 mm dish with the HMEC-1 monolayer and IRBC was then mounted into the flow chamber system. HBSS was infused at 1 dyne/cm^2^, and the number of adherent cells was counted at 30 sec intervals for a total of 4 min and the mean taken. The results suggest that while PMA stimulation did not increase IRBC adhesion under flow conditions, the adhesion of IRBC to PMA-stimulated HMEC-1 was significantly increased when the IRBC were allowed to bind under static conditions. Moreover, the enhancing effect of PMA was abrogated by pre-treatment of the HMEC-1 monolayers with RGD but not RAD. The increase in the number of adherent IRBC on PMA-stimulated monolayers was not associated with an increase in CD36, β_1_ integrin or ICAM-1 expression (Figure 10B).

## Discussion

The adhesion of *P. falciparum*-infected red cells to host endothelial cells in vital organs such as the brain and lung plays a fundamental role in the progression and outcome of the infection [42]. In this report, we showed for the first time by loss- and gain-of function assays that the integrin α_5_β_1_ may have a significant role in this pathological process on human microvascular endothelium. Our data suggests that in the resting state, α_5_β_1_ does not support adhesive interactions between IRBC and HDMEC. Upon IRBC adhesion to CD36, the integrin is either recruited passively as part of a molecular complex with CD36, or recruited actively to the site of IRBC attachment on CD36 ligation through phosphorylation of Src family kinases, a process that is Ca^2+^-dependent. Clustering of b_1_ integrin is associated with an increase in IRBC recruitment under flow conditions as well as an increase in adhesive strength after attachment on both unstimulated and TNF-α-stimulated endothelium. Conformational change of the integrin does not appear to play a role, as neither an activating antibody nor Mn^2+^ had any effect on IRBC adhesion. The binding of IRBC to clustered β_1_ could be inhibited by RGD and inhibitory antibodies to β_1_ and α_5_ integrins.

As adhesion molecule expression can be affected by cytokines in the microenvironment, we also tested if integrins would have a role in IRBC adhesion to TNF-α-stimulated HDMEC. We found that TNF-α did not affect the surface expression of α_5_ or β_1_, and the RGD peptide inhibited adhesion as on unstimulated endothelium. Moreover, anti-CD36 beads recruited β_1_ integrin on stimulated endothelium. Anti-ICAM-1 and RGD inhibited IRBC adhesion to a similar degree, but their effects were not additive, suggesting that α_5_β_1_ and ICAM-1 have a similar functional role in IRBC adhesion under flow conditions. This finding is consistent with our previous in vitro [3] and in vivo [4] observations that for IRBC that adhere to CD36, such as most clinical isolates from Thailand, ICAM-1 plays an important accessory role in increasing the number of adherent cells by reducing their rolling velocity. The relative contribution of integrins to IRBC cytoadherence compared to adhesion molecules that are upregulated or induced on stimulated endothelium suggested by our current findings will need to be confirmed with a large number of clinical parasite isolates with different binding phenotypes.

An association between integrins and CD36 has been described in several human cell types, including melanoma cells [21], microglial cells [16], platelets [43], and HDMEC [31]. In melanoma cells, the association between CD36 and b_1_ integrins requires the extracellular domain of the CD36 molecule. The association may occur within raft domains, since ectopic expression of CD36 increases the proportion of β_1_ integrins found within this fraction. In microglial cells, CD36, α_6_β_1_ and CD47 form a receptor complex for fibrillar β-amyloid, and antagonists specific for each component inhibits phagocytosis of β-amyloid to the same extent, suggesting that each component of the receptor complex is required but not sufficient for uptake of β-amyloid. In both platelets [43] and HDMEC [31], two distinct pools of CD36 in the cell membrane have been identified. One pool of CD36 is distributed in low-density lipid rafts and co-localizes with the Src family kinases. The other pool is in the high density soluble fraction that also contain β_1_ integrin, VEGFR-2, Syk and tetraspanins. Thrombospondin 1 (TSP-1) is also strongly detected in the high-density fraction, and participates in the formation of the VEGFR-2-Syk-CD36 complex that regulates angiogenesis. The 40% reduction of IRBC adhesion and adhesive strength by β_1_ integrin knock down may be mediated by this high density pool of CD36 in the cell membrane. Collectively, the evidence points to a close functional and subcellular association of CD36 with β_1_ integrin in multiple cell types.

The integrin α_5_β_1_ is one of 24 known members of the integrin family of adhesion molecules that are formed by noncovalent linkage of an α and β subunit with the ligand-binding ‘head’ region of the integrin being formed by both subunits [44]. Integrin α_5_β_1_ can exist in multiple conformational states, i.e. inactivated, intermediate activated, and fully activated. It can be activated by inside-out signaling or by non-physiological stimuli such as an activating antibody or extracellular Mn^2+^/Mg^2+^. The natural ligand of α_5_β_1_ is the extracellular matrix protein fibronectin (FN), a dimeric glycoprotein composed of two subunits each with multiple homologous domains named FNI, FNII, and FNIII. Optimal binding of FN to the integrin requires both the RGD motif present in FNIII domain 10 (FN10) and a synergy site located in FN9 [45]. The RGD motif binds to the β_1_ subunit while the synergy site interacts with α_5_. AFM studies have shown that FN with RGD deletion binds weakly to α_5_β_1_, while the force of detachment is only slightly less than wild type when the synergy site is mutated, and there is no enhancement of binding upon integrin activation [46]. These findings would support our results on the differential effect of β_1_ and α_5_ knock down on IRBC adhesion and adhesive strength. Interaction with both subunits appeared to be essential for IRBC adhesion under shear stress in the flow chamber assay. In the AFM experiments, IRBC and HDMEC were brought together mechanically at a constant rate and kept in contact by a constant force. In this situation, the interaction with α_5_ appeared dispensable, as evidenced by the normal force of detachment in α_5_ knockdown cells, and in cells pre-treated with the anti-α_5_ mAb JBS5. As endothelial cells are surrounded by an extracellular glycocalyx of approximately 30 to 50 nm in thickness that cannot be breached by either the bent (10 nm) or extended (20 nm) integrin forms [47], the initial attachment to CD36 may in addition to clustering lead to compression of the glycocalyx, bringing α_5_β_1_ integrin to close proximity of ligands on IRBC.

Several mechanisms that underlie the change in affinity or avidity of integrins for their ligands have been proposed [48]. The importance of clustering in promoting the avidity of integrin-ligand interactions, i.e. an increase in adhesiveness independently of integrin conformational changes, has been demonstrated by single molecule tracking. Using this technique, PMA was seen to induce a 10-fold increase in the lateral diffusion rate of LFA-1 in EBV transformed B lymphocytes [40]. The movement induced was random instead of directed, indicating that it was due to a release of the integrin from cytoskeletal attachment and subsequent free diffusion rather than a directional movement due to the application of forces. As a corollary, it would appear that the nonadhesive state of integrins is actively maintained by the cytoskeleton. The movement of LFA-1 was considered an important early step in the adhesion of monocytes to immobilized ICAM-1/E-selectin under flow conditions [41]. However, the bond formed by IRBC with clustered integrins on HMEC-1 induced by PMA appeared to be unable to withstand shear flow. Whether ligation by IRBC results in integrin activation and/or outside-in signaling that further modifies endothelial cell functions such as barrier function or proinflammatory mediator production remains to be determined.

Using an α_v_β_3_/α_v_β_5_-specific antagonist (cRGDfV) and an inhibitory mAb to α_v_β_3_, we were unable to confirm a role for α_v_β_3_ integrin in mediating cytoadherence on HDMEC. Nevertheless, α_v_β_3_ or other integrins may play an important role in IRBC adhesion to endothelial cells in other anatomical locations or other cell types, as already demonstrated for dendritic cells [27]. Integrins on the surface of platelets also play an active role in many physiological process, e.g. α_IIb_β_3_ in thrombus formation [49], and may quite likely participate in the interaction of IRBC and platelets.

The ligand(s) on IRBC that interacts with α_5_β_1_ integrin remains to be determined. The cytoadherent ligand PfEMP1 appears to be the most likely candidate, but ligands of host cell origin such as phosphatidylserine that is exposed by changes in membrane topography induced by an intracellular parasite may also play a part [50]. The presence of RGD motifs on PfEMP1 was noted in the initial reports on the cloning of the gene in the lab-adapted parasite clones MC and FCR3 [22], [23]. While RGD motifs did not occur in each protein sequence examined, their positions varied (DBL1-4) when they did occur. More recently, an analysis of seven *P. falciparum* genomes revealed that RGD motifs are overrepresented in highly conserved positions in the DBLα0 domain in close proximity to several cysteine residues [24]. This observation raises the interesting question of why the localization of RGD motifs on PfEMP1 is conserved in _1_a parasite protein that is otherwise highly structurally variant. Further studies into the process of ligand recognition between IRBC and integrins may shed light not only on IRBC adhesion to endothelial cells, but to other host cells such as monocyte/macrophages and platelets.

In summary, we have demonstrated that the integrin α_5_β_1_ acts synergistically with CD36 in mediating cytoadherence of IRBC to primary human microvascular endothelium. Together with our previous finding of IRBC-induced CD36 clustering and actin cytoskeletal rearrangement [6], a picture is emerging of the formation of a cytadherence synapse involving multiple adhesion molecules and ligand(s) on IRBC. This type of cooperative adhesive interactions may be of critical importance in enabling IRBC adhesion in the microcirculations. The ultimate goal for elucidating the molecules and processes involved in cytoadherence to primary endothelial cells is to develop rational treatments that could target key receptor molecules that come into play under physiological shear stress. Integrins are known to mediate immune cell recruitment and tumor cell migration that are associated with autoimmune diseases such as multiple sclerosis and different types of cancer respectively, and have become common therapeutic targets for these diseases [26]. As CD36 on different host cells may have both a protective and a pathological role against *P. falciparum* in the human host, anti-adhesive therapy targeted against its functional partners such as integrins with antibodies or small peptides rather than CD36 itself may provide a mechanism to decrease IRBC cytoadherence to microvascular endothelium while preserving the beneficial effects of this molecule against *P. falciparum*.

## Materials and Methods

### Ethics statement

The collection of *P. falciparum*-infected blood specimens was approved by the Ethics Committee of the Faculty of Tropical Medicine, Mahidol University, Bangkok, Thailand. Written informed consent was obtained from all patients and/or their legal guardians according to the Declaration of Helsinki. The collection of discarded foreskins for the isolation of endothelial cells and red blood cells from normal donors was approved by the Conjoint Ethics Board of Alberta Health Services and The University of Calgary, Alberta, Canada.

### Tissue culture and other reagents

Unless otherwise specified, all tissue culture and PCR reagents were obtained from Invitrogen Life Technologies Canada Inc. (Burlington, ON) and chemical reagents were purchased from Sigma-Aldrich Co. (St. Louis, MO). The Src-family kinase inhibitor PP1 and the inactive analog PP3 were purchased from Enzo Life Sciences International, Inc. (Plymouth Meeting, PA). Chemiluminescence HRP substrate was purchased from Millipore Corp. (Billerica, MA). Endothelial basal medium (EBM) was purchased from Lonza Walkersville, Inc. (Walkersville, MD).

### Antibodies and peptides

The following mAb were used: anti-human CD36 clone FA6-152 (Beckman Coulter Canada, Inc., Mississauga, ON); anti-human integrin β_1_ clones TDM 29 and TS2/16 (Millipore); FITC- and PE-labelled anti-human integrin β_1_ clone MEM-101A (Abcam, Cambridge, MA); anti-human α_5_ clones JBS5 (Millipore); anti-human ICAM-1 clone 84H10 (R&D Systems, Inc Minneapolis, MN); anti-human α_v_β_3_ clone 23C6 (Chemicon International); mouse IgG1 clone 11711 (R&D Systems); anti-phospho-Tyr418Src (BioSource; Invitrogen), anti-His-tag (His-probe (H-15)) (Santa Cruz Biotechnology Inc., Santa Cruz, CA); FITC goat anti-mouse IgG_1_ (Becton Dickinson, San Diego, CA); and Alexa Fluor 488 or 568 goat anti-mouse IgG_1_ antibodies and rhodamine-phalloidin (Molecular Probes, Invitrogen). Horseradish peroxidase (HRP)-conjugated secondary antibodies were purchased from Jackson ImmunoResearch Laboratories (West Grove, PA).

The RGD (H-Gly-Arg-Gly-Asp-Ser-Pro-OH) and RAD (H-Gly-Arg-Ala-Asp-Ser-Pro-OH) peptides were purchased from Calbiochem, EMD Bioscience Inc, La Jolla, CA. cRGDfV (cyclo Arg-Gly-Asp-D-Phe-Val and cRADfV (cyclo Arg-Ala-Asp-D-Phe-Val) were from Bachem Inc., Torrance, CA.

### Parasites

The majority of the experiments was performed with the parasite line 7G8 that binds to both CD36 and ICAM-1. The stock culture was shown to be free of mycoplasma contamination by RT-PCR (MycoAlert, Lonza Walkersville, Inc., Walkersville, MD). Frozen aliquots were thawed and cultured for 24 to 30 h at 37°C and 5% CO_2_ until the late trophozoite/early schizont stage as determined by light microscopy. IRBC cultures were used in single experiments and then discarded.

Experiments were also performed with cryopreserved clinical parasite isolates obtained from adult Thai patients with acute falciparum malaria at the Hospital for Tropical Diseases, Bangkok, Thailand [6].

### Microvascular endothelial cells

Primary human dermal microvascular endothelial cells were harvested from discarded neonatal human foreskins using 0.5 mg/ml Type IA collagenase (Roche Diagnostics, Indianapolis, IN) as described [3]. Harvested cells were seeded in 60 mm tissue culture dishes in endothelial basal medium (EBM) with supplements provided by the manufacturer. When cells were confluent, they were further purified on a magnetic bead separation column using CD31-coated beads (Miltenyi Biotec, Auburn, CA). Only cell preparations which were >95% positive for CD36 expression by flow cytometry were maintained for experiments. Experiments were performed with cells from passages two to five that were demonstrated to consistently support IRBC adhesion. HMEC-1, an immortalized cell line derived from HDMEC [29], was a kind gift of F.J. Candal at Emory University, Atlanta, Georgia. The cell line was maintained in EBM as for primary cells.

### Transduction of HMEC-1 with adeno-CD36-GFP

GFP-labeled CD36 was produced using the AdEasy adenoviral system (Stratagene, La Jolla, CA). GFP-labeled ICAM-1 was produced using the Virapower adenoviral expression system (Invitrogen). The selected recombinant was used to transfect HEK293 cells where deleted viral assembly genes were complemented in vivo. Harvested virus titers were adjusted to 1.0×10^10^ plaque-forming units (pfu) per ml. Viruses with the adeno-GFP construct were used as the control. Transduction was carried out using 1.0–2.0×10^7^ pfu/ml. The recombinant adenoviruses were routinely tested for the presence of endotoxin using the Kinetic QCL Limulus Amebocyte Lysate assay (Lonza, Walkersville, MD) and contained <0.3 endotoxin units/ml. Expression of CD36 and ICAM-1 on transduced cells was confirmed by both immunofluorescence microscopy and flow cytometry.

### Small interference RNA for β_1_ and α_5_ integrin

HDMEC were seeded in 35 mm tissue culture dishes at 2×10^5^ cells/dish and transfected 24 h later when the cells were 50 to 60% confluent. At the time of transfection the medium was aspirated and replaced with 1.0 ml of Optimem. The transfection mixture of 10 µl HiPerfect (Qiagen. GmBH, Hilden, Germany) and 20 nM siRNA for β_1_ integrin (Qiagen) or α_5_ integrin or 20 nM scrambled siRNA (All Stars Negative Control, Qiagen) was added in 100 µl of Optimem. Four hours after transfection, 1.0 ml of EBM was added to each dish. Monolayers were used for flow chamber studies 72 h after transfection. Cell lysates collected at the same time were used to confirm gene knockdown by Western blot analysis. For AFM experiments, transfected cells were trypsinized after 24 h and seeded on 25 mm glass coverslips (see below).

### PpMC-179 and antibody coated beads

The recombinant PpMC-179 protein representing the minimal binding domain of PfEMP1 (residues 88–267 of the CIDR domain of the parasite strain Malayan Camp) for CD36 [39] with a His_6_-tag on the C terminus was used to coat carboxyl functionalized polystyrene beads with a diameter approximating that of red blood cells (6.37±0.37 mm, Bangs Laboratories Inc., Fishers, IN) [6]. Beads were first covalently coated with an anti-His-tag antibody according to the instructions of the manufacturer. Prior to being used, the beads were washed in PBS and resuspended at a ratio of 1 µl of beads to 1 µg of PpMC-179 peptide in 20 µl of PBS for 1 h at room temperature. The presence of PpMC-179 on extensively washed beads was confirmed by Western blot.

Polystyrene beads were also coated with FA6-152, a mAb known to inhibit IRBC binding to CD36, or 84H10, a mAb known to inhibit IRBC binding to ICAM-1. One microgram of antibody in 10 µl PBS was added to 1 µl of beads for 2 h on a rocker and then let stand for 1 h. After washing in PBS, the beads were blocked with 100 µl of 0.1% BSA/PBS for 30 min. Beads were used within 48 h of preparation. The presence of antibody on the beads was confirmed by flow cytometry using goat-anti-mouse IgG-Alexa 488. Mouse IgG_1_-coated beads were used as controls.

### Confocal microscopy

HDMEC were seeded into μ-slideVI^0.4^ (Ibidi GmbH, Munich, Germany) at 2×10^4^ cells/chamber. When cells were 95% confluent (48 h), they were transduced with adenoviral GFP-CD36. For live cell imaging, MACS purified IRBC at 0.1% hematocrit were added to the chamber 24 hours after transduction, followed by the addition of FITC-labelled anti-β_1_ integrin. IRBC and HDMEC interactions were imaged in an enclosed, humidified chamber maintained at 37°C and 5% CO_2_. For experiments with antibody or peptide-coated beads, 0.5 µl of antibody- or peptide-coated beads resuspended in 60 µl HBSS were incubated with monolayers at 37°C and 5% CO_2_ for 30 min. After unbound beads were removed with HBSS, the monolayers were fixed with 1%PFA for 30 min at room temperature. They were blocked with 1%BSA and stained, or permeabilized with 0.2% Triton X-100 for 5 min prior to labeling with antibodies.

Quantification of β_1_ integrin clustering was performed by randomly selecting 3 microscopic fields at 60× magnification per condition per experiment as described [6]. Except for IgG_1_ or anti-His-tag coated beads, each field contained an average of 15 to 20 beads. Adherent beads associated with localized patches of fluorescence or discrete fluorescent rings were scored as positive. Beads that were partially visible in the field of view or rings that were present without beads were excluded. Each field was scored independently by 2 blinded observers. Results are expressed as per cent beads positive for protein recruitment.

### Flow chamber assay

IRBC-endothelial cell interactions at fluid shear stresses approximating those in the microvasculature were studied using a parallel plate flow chamber as described [3]. A 1% IRBC suspension was infused over confluent HDMEC monolayers at 1 dyne/cm^2^ that allowed us to optimally visualize the adhesive interactions in real time. Experiments were recorded and analyzed off-line. A rolling IRBC was defined as one which displayed a typical end-on-end rolling motion at a velocity of <150 mm/sec, compared to a centerline red blood cell flow rate of >1000 mm/sec, and a velocity of >150 mm/sec for non-interacting cells in close proximity to the endothelial monolayer. An adherent IRBC was defined as one which remained attached for >10 sec. Results were expressed as the number of adherent IRBC/mm^2^.

### Single cell force spectroscopy

Force spectroscopy was performed using a Nanowizard II atomic force microscope (AFM) equipped with a CellHesion Module (JPK Instruments, Berlin, Germany) as described [6]. The AFM was mounted on the stage of a Zeiss Axiovert 200 inverted light microscope (Carl Zeiss, Thornwood, NY) both of which were enclosed in a humidified chamber maintained at 37°C and 5% CO_2_. Force measurements were obtained using relative contact mode with a relative set point of 150 pN, extend/retract rates of 1.5 µm/s, Z-length of ∼50 µm, and a sample rate of 256 Hz. The baseline vertical offset was adjusted prior to every AFM reading and the software was set to correct for nonlinearity and hysteresis of the piezo. Analysis of data to determine the maximum detachment force was by software provided by JPK Instruments.

Force measurement experiments were performed with 2×10^4^ HDMEC seeded on one half of a 25 mm round glass coverslips pre-coated with 0.2% gelatin. At the time of confluence (48 h), the coverslips were loaded into a custom-built liquid cell that allowed the force measurements to be performed in fluid phase. The monolayers were washed twice with HBSS. A volume of 1 µl of IRBC at 0.5% hematocrit and 5 to 10% parasitemia was added to the half of the coverslip not seeded with HDMEC. The liquid cell was placed on the stage of the inverted microscope. At the start of the experiment, the cantilever that had been functionalized with dopamine hydrochloride was lowered onto an IRBC that was allowed to adhere for 60 sec before the cantilever with the adherent IRBC was moved to the monolayer and brought into contact with an endothelial cell.

In experiments in which inhibitors and antibodies were used, PP1 (10 µM) and PP3 (10 µM) were diluted from 1000× stock solutions in DMSO to the working concentration in culture medium and incubated with HDMEC monolayers for 30 min at 37°C. After removal of inhibitors, monolayers were washed 2× with HBSS before force measurements were carried out. For antibodies, the HDMEC monolayers were loaded into the AFM chamber, washed twice with HBSS, and then incubated with 200 µl of antibody at 5 to 10 µg/ml in HBSS for 30 min at 37°C. Prior to force measurements, the monolayers were washed 2× with HBSS.

### Statistical analysis

Statistical analysis was performed using GraphPad Prism (version 4, GraphPad Software Inc.). Data are expressed as mean ± SEM unless otherwise stated. Data from two groups were compared using Student's 2-tailed t-test for paired samples unless otherwise specified. p values of 0.05 or less were considered statistically significant.

## Supporting Information

Figure S1 **Adhesion molecule expression by HDMEC.** (A) Flow cytometric analysis and (B) confocal immunofluorescence microscopy of CD36, α_5_, β_1_ and α_v_β_3_ surface expression. Cells for flow cytometry were harvested from 35 mm dishes by trypsinization. One hundred microliters of cell suspension were incubated with primary antibody for 30 min at 4°C. After washing, a FITC-labelled goat anti-mouse IgG_1_ was added for 30 min at 4°C. For microscopy, PFA-fixed monolayers in ibidi chambers were stained with a primary antibody overnight at 4°C. After washing, an Alexa 488-labelled goat anti-mouse IgG_1_ was added for an hour at room temperature. All images were taken on an Olympus IX81 inverted confocal microscope (Center Valley, Pa) with Fluorview 1000 acquisition software using a PlanAPO 60× N.A. 1.42 oil immersion objective. Results shown for both (A) and (B) are representative of at least 3 experiments.
(TIF)
