# Supplementary figures and images for: CD36 Recruits α5β1 Integrin to Promote Cytoadherence of P. falciparum-Infected Erythrocytes
Source: PLoS Pathog. 2013 Aug 29;9(8):e1003590. doi: 10.1371/journal.ppat.1003590 (PMC3757042; doi:10.1371/journal.ppat.1003590)

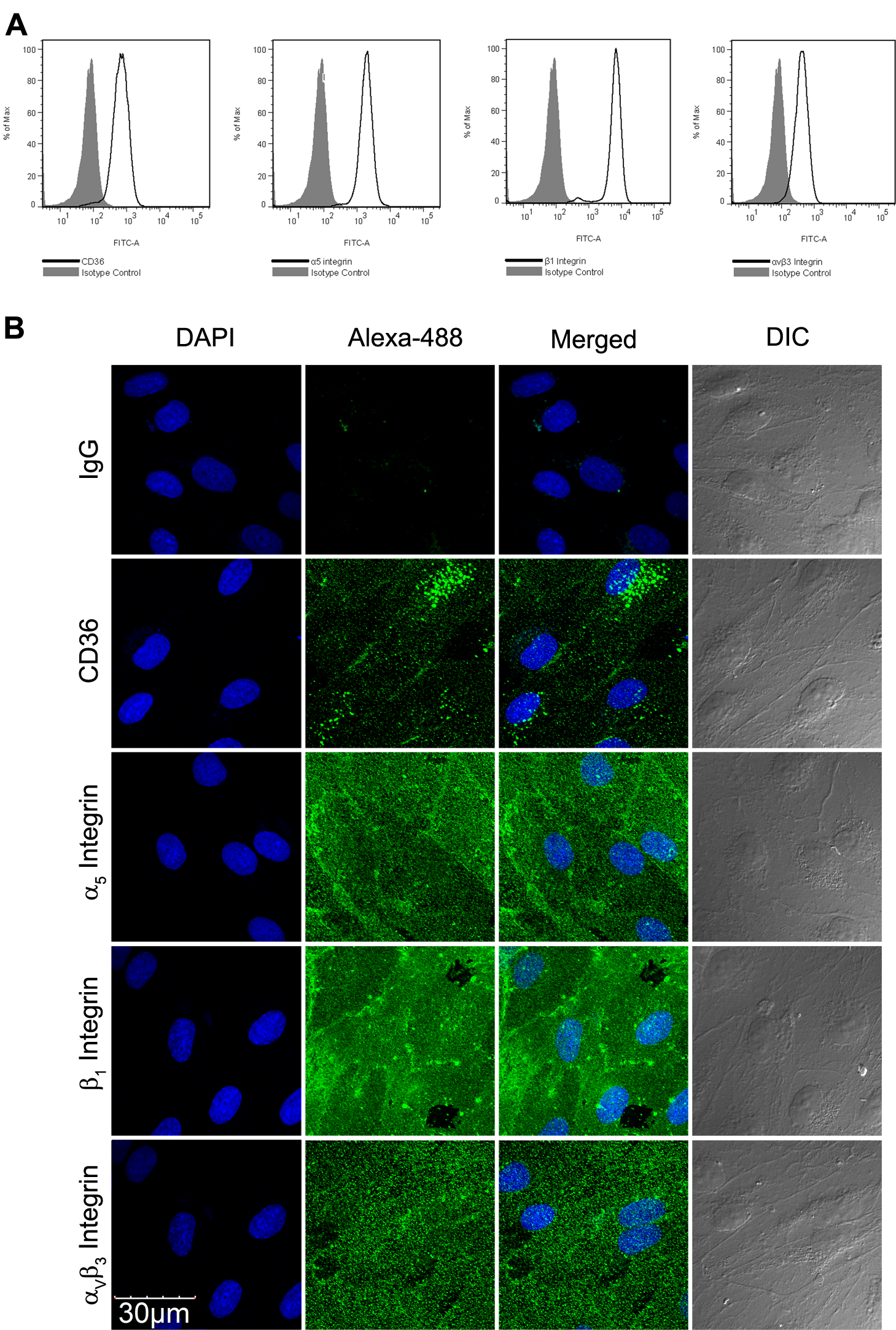

Supplement: Figure S1 — Adhesion molecule expression by HDMEC. (A) Flow cytometric analysis and (B) confocal immunofluorescence microscopy of CD36, α5, β1 and αvβ3 surface expression. Cells for flow cytometry were harvested from 35 mm dishes by trypsinization. One hundred microliters of cell suspension were incubated with primary antibody for 30 min at 4°C. After washing, a FITC-labelled goat anti-mouse IgG1 was added for 30 min at 4°C. For microscopy, PFA-fixed monolayers in ibidi chambers were stained with a primary antibody overnight at 4°C. After washing, an Alexa 488-labelled goat anti-mouse IgG1 was added for an hour at room temperature. All images were taken on an Olympus IX81 inverted confocal microscope (Center Valley, Pa) with Fluorview 1000 acquisition software using a PlanAPO 60× N.A. 1.42 oil immersion objective. Results shown for both (A) and (B) are representative of at least 3 experiments. (TIF) [file ppat.1003590.s001.tif]
